# The State of Sexual Health in United States of America in 2024: Results from the World Health Organization’s Sexual Health Survey (SHAPE)

**DOI:** 10.1080/0092623X.2026.2646636

**Published:** 2026-03-30

**Authors:** Jessie V. Ford, Eli Coleman, Leonor de Oliveira, Ryan L. Rahm-Knigge, Kristen P. Mark

**Affiliations:** a Sociomedical Sciences, Columbia University, New York, NY, USA;; b Eli Coleman Institute for Sexual and Gender Health, Department of Family Medicine and Community Health, University of Minnesota Medical School, Minneapolis, Minnesota, USA

## Abstract

A cross-sectional online survey conducted in July 2024 assessed sexual health in the U.S. using the World Health Organization’s Sexual Health Assessment of Practices and Experiences (SHAPE) questionnaire. The study included 2555 adults aged 18–94 years, recruited through Prodege’s opt-in research panel to reflect U.S. demographic diversity. Measures captured sexual knowledge, communication, attitudes, behaviors, use of sexual health services, and adverse outcomes, aligned with four sexual health framework objectives. Results showed high sexual wantedness (89%) and pleasure (87%) during the most recent sexual encounter but moderate overall satisfaction (56%). Gender differences emerged: women and gender-diverse participants reported lower pleasure, higher sexual violence exposure, and reduced safety in public spaces compared to men. Testing for HIV and other STIs was suboptimal, with 50% and 47% of participants never tested, respectively. Adverse outcomes included self-reported unintended pregnancy (40%), adolescent pregnancy (19%), and HIV prevalence of 3%. Sexual health communication was more common with partners (49%) than healthcare providers (31%). These findings reveal persistent inequities and limited progress toward equitable sexual health in the U.S. Strengthened national strategies, comprehensive and inclusive sexuality education, expanded access to competent and inclusive care, and improved surveillance are urgently needed to advance sexual health for all populations.

## Introduction

Sexual Health is defined by the World Health Organization (WHO) as a state of physical, emotional, mental and social wellbeing in relation to sexuality that goes beyond the absence of disease, dysfunction, or infirmity. The WHO working definition goes on to emphasize the requirement of “a positive and respectful approach to sexuality and sexual relationships, as well as the possibility of having pleasurable and safe sexual experiences, free of coercion, discrimination and violence” (World, 2006). The definition states that in order to attain and maintain sexual health, the “sexual rights of all persons must be respected, protected and fulfilled”. This pioneering acknowledgement of holistic sexuality paves the way for researchers and policy makers to operationalize positive sexuality as an important indicator of population health.^1^

Despite the ambitious vision embedded in the WHO definition, public health efforts in the U.S. have historically addressed sexual health through narrow, disease-specific initiatives rather than adopting a broad, integrated framework. Programs focused on family planning or STI/HIV prevention often operate in silos, driven by topic-specific funding and organizational structures. This fragmented approach has led to repeated calls for a national strategy that comprehensively addresses sexual health. One of the earliest major efforts in this direction was *The Surgeon General’s Call to Action to Promote Sexual Health and Responsible Sexual Behavior* in 2001 (Satcher, 2001), which defined sexual health in a similar way to the WHO definition and marked the U.S. government’s formal recognition of the importance of a comprehensive approach to sexual health.

Adding to this challenge, recent legal and policy developments in the U.S. have created a challenging environment for advancing comprehensive sexual and reproductive health (SRH). In the post-*Dobbs* landscape, abortion access has been substantially constrained, and restrictive reproductive health policies frequently cluster with legislative efforts affecting gender-affirming care, LGBTQ+ inclusion, and sexuality education (Gutiérrez et al., 2024; Wang, 2025). States adopting the most restrictive reproductive health policies also tend to exhibit limited public health infrastructure to support equitable access to care, underscoring the need for coordinated public health strategies to protect sexual health and well-being (Madden et al., 2024).

In recent years, various policy documents and initiatives have acknowledged the importance of sexual health. These include Healthy People 2030, reports by the National Academies of Sciences, Engineering, and Medicine (NASEM), the White House National Strategy on Gender Equity and Equality, the World Health Organization’s (WHO) Global STI Strategy, and several Center for Disease Control and Prevention (CDC)-led initiatives (CDC, 2022; HHS, 2021; NASEM 2021; ONAP, 2021; The White House, 2022; WHO 2017; Workowski et al., 2021). However, while these frameworks reference sexual health, they tend to focus on specific problems (e.g., reducing unintended pregnancies or STI rates) and stop short of promoting a truly holistic or integrated approach.

Advocates for a broader vision of sexual health—such as Satcher (2001), Satcher et al. (2015), Swartzendruber and Zenilman (2010), Ivankovich et al. (2013) and others—have argued for a model that considers sexual well-being throughout the lifespan. In response, a comprehensive sexual health framework was proposed in 2017 (Ford et al., 2017). This model emphasizes wellness, positive and respectful relationships, and the recognition of sexual health as a key component of overall health. It includes four long-term objectives:

1. Increase knowledge, communication, and respectful attitudes
2. Increase healthy, responsible, and respectful sexual behaviors and relationships
3. Increase use of high-quality, coordinated educational, clinical, and preventive services
4. Decrease adverse sexual health outcomes

To track progress toward these objectives, the framework proposes the use of specific sexual health indicators. These would provide public health agencies and stakeholders with standardized tools to monitor outcomes and apply evidence-based interventions across the lifespan. Similar scorecard models and indicator systems have been implemented in other countries, such as the United Kingdom (GOV.UK, 2022) and in international efforts led by the United Nations Population Fund (UNFPA, 2022, 2024; WHO, 2020). Several nations—including Australia, Canada, Belgium, France, Germany, and the Netherlands (De Graaf & Van Santen, 2014; Fisher et al., 2019; Matthiesen et al., 2021; Public Health Agency of Canada, 2012)—have also developed robust national sexual health surveys. These surveys provide valuable data for shaping policies, evaluating programs, and understanding trends at both national and local levels.

At present, a consistent national survey over time that provides a snapshot of Americans’ sexual health has not been conducted. Instead, measures of sexual health (e.g., STI testing, access to sexual health services) have been embedded in national surveys (e.g., the National Survey of Family Growth (NSFG)) and disease surveillance systems or conducted through private funding sources (e.g., the National Survey of Sexual Health and Behavior (NSSHB)). At present, many of these datasets have restricted access or are limited to specific populations (e.g., reproductive age).

In 2023, Ford, Ivankovich, and Coleman compiled existing data items from various available sources and provided a U.S. sexual health scorecard.(Ford et al., 2022) However, many of the resulting indicators were imperfect proxies for the constructs the authors sought to measure, reflecting limitations in the underlying surveys. For example, there were no measures of communication about sexual health with healthcare providers, and attitudinal measures were largely restricted to general attitudes toward same-sex sexual behavior. Key dimensions of sexual well-being—such as sexual pleasure, satisfaction, and whether sexual activity was wanted—were not measured; instead, available indicators relied primarily on happiness in marriage as a rough substitute. Additionally, no national indicators captured experiences related to safety or stigma, abortion access, fertility, or stillbirth outcomes. Finally, the available national data excluded older adults, as many indicators were drawn from surveys such as the National Survey of Family Growth, which largely ends data collection at age 49.

### Present study

In this study, we use a sexual health framework to analyze 2024 data collected on the state of sexual health in the U.S. The WHO’s publication of the Sexual Health Assessment of Practices and Experiences (SHAPE) (WHO 2023) questionnaire provided a new tool for collecting sexual health data and producing a U.S. scorecard. While one Portuguese study (Patrão et al., 2025) evaluated the implementation of SHAPE, demonstrating its validity and feasibility, it did not report sexual health outcomes. This article is the first to present SHAPE data from a country (the U.S.), including knowledge, attitudes, behaviors, services, and outcomes, enabling comparisons at a global level. This current analysis highlights the state of sexual health in the U.S. with the aim of identifying needs and implications to improve population health.

## Methods

### Survey procedure

Participants were recruited through Protege, a market research company that has the capacity to recruit large samples that include a broad range of U.S. demographic groups using their opt-in panels. Protege screens respondents based on eligibility criteria: age 18 or older, U.S. residency, and English fluency. To ensure data quality, Prodege employs verification and fraud detection tools, including dual opt-in, account checks, GEO-IP tracking, bot traps, behavioral analysis, pattern detection, and continual monitoring for suspicious activity. These tools ensure quality control that detects bots, survey farms, and multi-tasking survey respondents.

### Informed consent and ethical approval

Participants were recruited via the Prodege platform in July 2024 and completed the survey online via QualtricsXM. Informed consent was obtained electronically and anonymously. The consent form outlined study details, including risks, benefits, and contact information. Participants were paid between $0.50 and $2.00 USD for completing the survey, with the exact amount depending on the specific demographic targeting criteria. The survey was made available to eligible panel members through their personalized dashboard upon logging into their Prodege accounts. Study procedures were approved by the Ethics Committee of [University of Minnesota]. Survey data were screened for duplicates, invalid or inconsistent responses, and missing data. Cases that did not complete additional required measures were excluded. Of the 2,902 initial respondents, 463 were removed, resulting in a final analytic sample of 2,555 English-speaking U.S. adults ages 18 to 94 (M = 47.26, SD = 16.91).

### What is the SHAPE?

The Sexual Health Assessment of Practices and Experiences (SHAPE) questionnaire, developed by the World Health Organization (WHO) between 2019 and 2023, is a standardized tool designed to assess sexual practices, behaviors, and health outcomes across global populations. Aligned with WHO’s comprehensive definition of sexual health, SHAPE captures both positive and adverse outcomes and aims to improve the collection and comparability of sexual health data internationally. SHAPE was created through a rigorous global consultative process, including technical consultations, open calls for researcher input, and expert reviews. The questionnaire was refined using cognitive interviews and implemented in three waves across 19 countries of varying income levels. Its final form consists of 75 items divided into six modules: personal and health information, sexual health outcomes, sexual biography, sexual practices, social perceptions and beliefs, and sociodemographic data.

In this analysis, we did not use the full set of 75 SHAPE items. Rather we selected those SHAPE indicators most aligned with the aforementioned comprehensive sexual health framework, focusing on knowledge, communication, behavior, access to services, and outcomes. Of note, the SHAPE did not include any items on communication. Therefore, we supplemented the SHAPE with two additional items from an existing CDC survey (CDC 2025) related to comfort talking about sexual health with healthcare providers and sexual partners. Table 1 includes the specific SHAPE items used to measure each sexual health objective. Of note, Responses of *“Don’t know”* or *“Prefer not to say”* were treated as missing in all analyses

## Results

As shown in Table 2, our sample for the U.S. SHAPE included a total of 2,555 English speaking U.S. residents aged between 18 and 94. The sample included more self-identified women than men (56% vs. 43%). For sexual orientation, 71% of the sample identified as straight (heterosexual). Nearly half (45.3%) reported that they were currently married. Approximately three-fourths (76%) of the sample self-identified as white. Over half the sample reported a college education or higher (53%). While the study sample was relatively large and included a broad range of U.S. demographic groups, it was not fully representative of the national population (see limitations for further discussion). Below we present results of the state of sexual health based on this sample.

### Objective 1: Increase knowledge, communication, and respectful attitudes regarding sexual health

As shown in Table 3, we found that 78% of our sample knew that sex education does *not* lead people to start having sex earlier. Although this measure does not fully reflect actual sexual health knowledge, it served as the best available proxy to measure knowledge in this dataset. These percentages were slightly higher for women (82%) and other gender identities^2^ (88%) compared to men (73%).

As shown in our measures (Table 1), we supplemented the SHAPE with existing two communication questions. We found that 49% of the sample was “completely comfortable” talking to sexual partners about their sexual health (50% men; 48% women; 55% other gender identities). A lower percentage of the population (31%) reported being “completely comfortable” talking to healthcare providers about their sexual health (34% men; 30% women; 23% other gender identities).

In contrast to knowledge and communication, the SHAPE had extensive measures of attitudes. Four attitudinal measures related to sexual health (personal views toward sex between men, gendered sex drives, abortion, and contraceptive use) were included in the survey. As shown in Table 3, 70% of people agreed that consensual sex between men is ok (67% men; 71% women; 96% other gender identities). Only 25% of the sample shared the personal view that “Men naturally have more sexual needs than women” (25% men; 26% women; 8% other gender identities). Forty-eight percent of the total sample supported the idea of an abortion for any reason (44% men; 49% women; 77% other gender identities). The vast majority–78% of participants—strongly agreed that it is “okay” to use contraception (72% men; 82% women; 92% other gender identities).

### Objective 2: Increase healthy, responsible, and respectful sexual behaviors and relationships

Four indicators were selected to examine healthy, responsible, and respectful sexual behaviors. For sexual debut, we found that 47% of the sample had sex before age 18 (45% men; 49% women; 44% of other gender identities). Among those who recently had sex, 60% of participants did not use any precautions against STIs or unintended pregnancies during their last sexual encounter (57% men; 62% women; 59% other gender identities).^3^ The vast majority (87%) of the sample described their most recent sexual encounter as very pleasurable or pleasurable (93% men; 82% women; 86% other gender identities). Satisfaction rates were moderate with 56% of the sample reporting being very satisfied or satisfied with their sex life in the last year (57% men; 56% women; 46% other gender identities).

### Objective 3: Increase use of high-quality, coordinated educational, clinical, and other preventive services that improve sexual health

As shown in Table 3, 50% of the sample had never been tested for HIV (53% men; 48% women; 35% other gender identities). Likewise, 47% of the sample had never been tested for STIs other than HIV (50% men; 45% women; 37% other gender identities). The abortion rate among those who had been pregnant or gotten someone pregnant was 29% overall (23% men; 32% women; 39% other gender identities).

### Objective 4: Decrease adverse health outcomes, including HIV/STIs, viral hepatitis, unintended pregnancies, sexual violence, sexual dysfunction, and cancers in reproductive tracts

The HIV prevalence in our sample was 3% overall (8% among men and less than 1% for women; 1% for other gender identities. An estimated 17% had ever received treatment for an STI other than HIV (17% men; 16% men; 23% other gender identities). Our sample had a 40% rate of unintended pregnancy (40% men; 39% women; 81% other gender identities). The adolescent pregnancy rate (under age 20) was 19% (11% men; 23% women; 14% other gender identities).

Around one quarter (27%) of people report having been forced or frightened into doing something sexually (12% men; 37% women; 56% other). Only 16% of the sample reported feeling not at all safe/somewhat unsafe outside their home from sexual assault (6% men; 22% women). This number was higher for other gender identities (39% reported feeling unsafe). In contrast, the vast majority of people reported high levels of sexual wantedness. Approximately 89% of the sample described their last sexual experience as wanted (91% men; 87% women; 90% other gender identities).

In our sample, 3% reported having had a stillbirth (4% men and 3% women; 7% other gender identities). Fertility difficulties were documented among 15% of the sample (12% men; 18% women; 12% other gender identities).

## Discussion

### Comparison with previous data on sexual health

Compared to national data from five years ago—including data from the NSFG, CDC, Guttmacher Institute, NSSHB, Gallup, Pew, and the General Social Survey (GSS)—the current findings reveal both progress and persistent inequities in sexual and reproductive health. We note that our sample includes a higher proportion of sexual minority participants than most national surveys, so comparative figures are provided only as contextual reference points, not benchmarks, allowing interpretation while remaining cautious about sample and cross-study differences. STI testing in our sample (47%) was slightly above national estimates (~40%). HIV testing (50%) in our sample was higher than national estimates (~40%), yet still not in line with CDC recommendations that everyone aged 13–64 be tested for HIV at least once (HIV.gov, 2019; Kaiser Family Foundation, 2011). HIV prevalence was significantly higher in this sample (3% overall, 8% among men) than prior national estimates (0.3%–0.5%) (CDC, 2025), likely due to an overrepresentation of high-risk groups such as men who have sex with men in our sample. Similarly, STI treatment rates were elevated (17% overall; 23% among gender-diverse individuals) compared to previous estimates of 10%–13% (McQuillan et al., 2018; Workowski et al., 2021), suggesting either higher risk or improved access to services.

Reproductive health patterns show mixed trends. While the overall unintended pregnancy rate (40%) aligns with past national levels, (Bearak et al., 2022; Rossen et al., 2023) adolescent pregnancy was almost double the national average (19% vs. <10%) (HHS, 2025). Stillbirth (3%) and fertility difficulty rates (15%) were also higher than previous estimates (1% and 10%–12%, respectively) (NCHS 2025a, 2025b), possibly reflecting growing health disparities or differences in sample composition.

Sexual safety remains a concern. Reports of lifetime sexual violence and feeling unsafe in public places were higher among women (37% and 22% respectively) and gender-diverse individuals (56% and 39% respectively) compared to men. These numbers echo other estimates (Black et al., 2011; Zhang Kudon et al., 2024) and may relate to broader gender inequality and discrimination based on gender.

Positively, 89% of participants described their last sexual experience as wanted and 87% described their last sexual experience as pleasurable or very pleasurable. These numbers were highest for men. These estimates align with other national surveys (Carter et al., 2019; Ford et al., 2022). In contrast, 56% of participants reported sexual satisfaction in the past year suggesting some complexity in how pleasure relates to satisfaction.

Attitudinally, the sample reflects growing acceptance: 71% supported same-sex relationships (up from 66% to 69% in national estimates (Brenan, 2024; Pew Research Center, 2021; Twenge & Blake, 2021), 25% rejected traditional gender norms around sexual needs (versus 40% previously) (Horowitz & Parker, 2024), and 48% supported abortion rights (versus 40% (Elder et al., 2024). While comfort discussing sexual health with partners was higher than national estimates (49% vs. 40%), conversations with healthcare providers remain challenging—particularly for non-cisgender individuals (Zhang et al., 2020).

### Notable findings on the state of sexual health in the U.S

First, the data suggest some gender differences across several outcomes that may reflect broader social and relational dynamics. For example, women reported somewhat greater hesitancy than men in discussing sexual health with providers or partners. Women and individuals of other gender identities also reported feeling less safe outside their homes and experienced higher rates of sexual violence compared to men. While most participants across all genders described their most recent sexual experience as pleasurable, men reported the highest rates of most recent pleasurable experience.

Another notable finding concerns how people label their sexual experiences. Although most participants described their most recent sexual encounter as wanted, lifetime reports of sexual violence remained high. This apparent contrast should not be interpreted as a contradiction. Instead, it may reflect, speculatively, growing awareness of sexual consent and reduced stigma around reporting harmful experiences. The simultaneous reporting of wanted sex and past non-consensual experiences could suggest that more individuals feel able to acknowledge both positive and harmful sexual experiences. While continued efforts to prevent sexual violence remain essential, it is tentatively encouraging that people may be more willing to recognize and report these experiences.

### Opportunities for improvement of sexual health in the U.S

While the data reveal some promising trends, including many people reporting that sex was wanted and a good number feeling able to talk to partners about sexual health, the broader picture is far less optimistic. Rates of sexual satisfaction are underwhelming, with a little over half of respondents reporting being satisfied. There are also major gaps in knowledge, access, and outcomes: STI and HIV testing remain low; comfort talking with providers is low, unintended pregnancy rates and fertility issues are persistently high; and many report lifetime STI diagnoses or frequent unprotected sex. These patterns point to a troubling disconnect between public health goals and the lived realities of people in the U.S. Stigma appears to continue around sexual health limiting open conversations, especially with healthcare providers.

Taken together, these findings offer a nuanced and urgent portrait of sexual health in the United States—one marked by persistent disparities, limited satisfaction, and significant gaps in care and education. Gender-based inequities remain deeply entrenched, with women and individuals of marginalized gender identities reporting less safety, less pleasure, and less comfort discussing sexual health, while also bearing a disproportionate burden of sexual violence. At the same time, the apparent contradiction between high rates of “wanted” sex and continued reports of non-consensual experiences suggests a growing public awareness of sexual consent and a decline in stigma—an encouraging signal of cultural change.

Yet these positive shifts exist alongside widespread gaps in access to sexual health services, poor testing rates for STIs and HIV, and a troubling disconnect between public health ideals and lived realities. To truly improve sexual health outcomes in the U.S., we must move beyond documenting the problems—we must act.

In order to address inequities and advance the sexual health of the U.S. population, a set of long-standing, evidence-based strategies must finally be implemented. These are not novel proposals; they have been articulated across numerous policy documents over the past two decades. The challenge now lies not in identifying solutions, but in committing to enacting them.

### Establish a national sexual health strategy

Much like existing national plans for HIV or mental health, this strategy should be coordinated at the federal level and grounded in the WHO definition of sexual health. It must promote awareness, invest in research, and emphasize a positive, holistic approach to sexual well-being—shifting the focus from disease avoidance to one of agency, consent, pleasure, and healthy relationships across the life course. This vision must include all people, regardless of age, gender, sexual orientation, race/ethnicity, or socioeconomic status.

We recognize that achieving these goals in today’s U.S. context is particularly challenging. Funding for sexual and reproductive health has been reduced in many areas, access to abortion and gender-affirming care is increasingly restricted, and broader anti-rights movements—including intensified debates over sex education, contraception, and LGBTQ+ inclusion—pose significant barriers. Even so, these objectives remain important and valid. In fact, the current environment underscores the urgency for public health officials, policymakers, and advocates to seriously consider and act on a comprehensive sexual health strategy. Addressing these challenges directly is essential to protect and promote the sexual health and well-being of all communities, particularly those most marginalized.

### Ensure access to comprehensive sexuality education

Mandating inclusive, age-appropriate, and medically accurate sexual education in all U.S. schools is foundational. Such education should cover critical topics like consent, gender identity, sexual orientation, pleasure, contraception, and digital safety. Education should also extend beyond youth and schools to include adults, caregivers, and community-based programs that foster sexual health literacy at all stages of life.

### Provide equitable access to inclusive, competent care

This includes expanding sexual and reproductive health services such as contraception, abortion, STI/HIV testing and treatment, gender-affirming care, and counseling for sexual function and trauma. Health providers must be trained in cultural humility, trauma-informed practices, and lifespan sexual health to ensure all individuals feel affirmed and respected in care settings. Public awareness campaigns can help normalize conversations about sexuality and reduce stigma at the societal level.

Additionally, the low rates of STI and HIV testing, combined with high rates of unintended and adolescent pregnancy, point to systemic barriers in preventive and reproductive health services. Clinicians play a crucial role in closing these gaps by normalizing sexual health screening and counseling as routine aspects of primary and mental health care. Expanding access to competent sexual healthcare including sexual educators, counselors and therapists—particularly in underserved and marginalized populations—can improve detection, prevention, and patient outcomes.

### Strengthen data collection and surveillance systems

Regular, comprehensive, population-based surveys—such as the SHAPE or models like the U.K.’s NATSAL—should be institutionalized. They should also be expanded. For instance, more countries need to use the SHAPE and publish these data for cross-country comparison. These data must be made accessible to communities, researchers, and policymakers to guide interventions and promote accountability. At the same time, the SHAPE could be expanded to better capture sexual health knowledge, receipt of sexuality education, communication, sexual dysfunction, and structural determinants such as stigma and gender inequality. Without these indicators, our understanding remains partial. Ultimately, to fully understand and promote sexual health, data must encompass pleasure, function, and satisfaction, alongside detailed demographic measures that reveal and help correct inequities.

### Address sexual health inequities

Data alone is not enough—we must use it to tackle the structural and social determinants that drive disparities. These include racism, sexism, homophobia, poverty, lack of education, housing insecurity, and the criminalization of sexuality. A comprehensive sexual health agenda must work across systems to reduce these root causes and ensure equitable sexual health outcomes for all, including rural and other underserved populations.

Ultimately, improving sexual health in the U.S. requires political will, cross-sector collaboration, and an unwavering commitment to justice, equity, and human dignity. Only by advancing this multifaceted agenda can we close the gap between aspiration and reality—and ensure that every person has the opportunity to experience sexual health, safety, and well-being throughout their life.

### Limitations

Several limitations should be considered when interpreting this study’s findings. The data were self-reported and cross-sectional, collected via an online opt-in panel. This introduces potential for recall bias, social desirability bias, and underreporting—especially on sensitive topics like sexual violence, abortion, and HIV status. The design also prevents causal inference or assessment of changes over time.

While the study sample was relatively large and included a broad range of U.S. demographic groups, it was not fully representative of the national population. The sample included only English-speaking U.S. adults over age 18, potentially excluding non-English speakers and adolescents and underrepresenting marginalized groups with unique barriers to sexual health services. The sample was overrepresented by females (56%) and older adults (aged 60 and above; 28.4%), while younger adults (aged 18–24) were underrepresented. There was also an overrepresentation of sexual and gender minorities. In terms of race and ethnicity, Hispanic/Latinx individuals were underrepresented (11%), whereas White (76%) and American Indian/Alaska Native (3.1%) participants were slightly overrepresented. The educational attainment of the sample was substantially higher than the national average, with 53% holding a college degree or higher (U.S. Census Bureau, 2024).

The opt-in nature of the panel may also lead to selection bias, with those more engaged in sexual health issues more likely to participate. Additionally, while we compare SHAPE survey results to existing U.S. data, these comparisons are limited by differences in methodology, populations, and measurement tools. Many national estimates used probability-based samples via in-person or telephone interviews, whereas our survey relied on self-administered online responses. These differences may affect both the reliability and comparability of findings.

Because of these demographic discrepancies and methodological issues, generalizing findings from this sample to the broader U.S. adult population should be done with caution. Certain groups—particularly younger adults, males, Hispanic/Latinx individuals, and those with lower educational attainment, non-English speaking or with other barriers for completing an online survey—are underrepresented, which may lead to results that reflect a somewhat skewed demographic profile (older, more educated, more female, and more sexual and gender minority participants) rather than the general U.S. population.

## Conclusion

This study describes the results of a national sexual health survey which could be used as a stepping stone for monitoring sexual health in the U.S. in a more robust, systematic and longitudinal manner. Based on these data, the current state of sexual health in the U.S. reveals a mix of progress and persistent gaps. While some individuals report positive experiences—such as wanted sex and improved communication with partners—significant disparities remain. Women and gender-diverse individuals face higher rates of sexual violence, lower sexual pleasure, and less comfort discussing sexual health, all of which point to deeply rooted gender inequities. Despite growing awareness of consent and reduced stigma around reporting negative sexual experiences, the disconnect between public health goals and lived realities remains stark, underscored by low STI/HIV testing rates, high unintended pregnancy, and discomfort discussing sexual health with providers.

To close these gaps, a national commitment is urgently needed. This includes establishing a comprehensive National Sexual Health Strategy aligned with the WHO framework, mandating inclusive and accurate sexuality education across the lifespan, expanding access to culturally competent and affirming care, and investing in robust, equity-focused data systems. These strategies—long recommended in public health policy—must now be enacted with urgency. Reducing disparities and advancing sexual health for all will require sustained investment, political will, and coordinated efforts to address the broader social determinants that shape people’s ability to experience safe, satisfying, and equitable sexual lives.

## Figures and Tables

**Table 1.** SHAPE survey items used to measure sexual health objectives.

Domain	Measure	Question/item	Response options	Reported response
**1) Knowledge, Attitudes, and Communication**	Knowledge	*“Teaching sex education in school does not lead to young people having sex earlier”*	Yes/No/Prefer not to say	Yes
	Communication	*“Are you comfortable talking to any healthcare providers about your sexual health?”*	Not at all comfortable/Somewhat uncomfortable/Somewhat comfortable/Completely comfortable	Completely comfortable
	Communication	*“Would you say you are comfortable talking to your sexual partners about your sexual health?”*	Not at all comfortable/Somewhat uncomfortable/Somewhat comfortable/Completely comfortable	Completely comfortable
	Attitudes: Sex between men	*“Sex between two consenting adult men is wrong/okay/Prefer not to say”*	Wrong/Okay/Prefer not to say	Okay
	Attitudes: Men’s sexual needs	*“Which of these statements is closest to your personal view? Men naturally have more sexual needs than women/Women naturally have more sexual needs than men/I don’t agree with either/Prefer not to say”*	Men more/Women more/Neither/Prefer not to say	Men more
	Attitudes: Abortion	*“Which of these statements is closest to your personal view? It is okay for someone to have an abortion for any reason/Only under certain circumstances/Always wrong/Prefer not to say”*	Any reason/Certain circumstances/Always wrong/Prefer not to say	Any reason
	Attitudes: Contraceptive acceptance	*“It is okay for someone to use a contraceptive method/family planning to avoid or delay pregnancy”*	Strongly agree/Agree/Disagree/Strongly disagree/Prefer not to say	Strongly agree
**2) Sexual Behaviors and Relationships**	Early sexual debut	*“How old were you the first time you had sex with someone?”*	Numeric age/Don’t know/Prefer not to say	% < 18
	Protection	*“What, if any, precautions against pregnancy or HIV/STIs did either of you take the most recent time you had sex? (Choose all that apply)”*	No precautions/Male condom/Female condom/Dental dam/PrEP/Birth control/Morning after pill/IUD/Cap/Injections/Implant/Spermicides/Withdrawal/Calendar method/Sterilized/Other/Don’t know/Prefer not to say	No precautions
	Sexual pleasure	*“How pleasurable did you find the most recent time you had sex?”*	Very pleasurable/Pleasurable/Neutral/Unpleasurable/Very unpleasurable/Prefer not to say	Very pleasurable or pleasurable
	Sexual satisfaction	*“In general, how satisfied have you been with your sex life in the last year?”*	Very satisfied/Satisfied/Neutral/Dissatisfied/Very dissatisfied/Prefer not to say	Very satisfied or satisfied
**3) Education and Service Use**	Education or training	*Not measured by SHAPE*	N/A	No data reported
	Vaccines	*Not measured by SHAPE*	N/A	No data reported
	HIV testing	*“When, if ever, were you last tested for HIV? Was it…”*	Within the last year/More than 1 year ago/Never/Don’t know/Prefer not to say	Within the last year or more than 1 year ago
	STI testing	*“Aside from HIV, when, if ever, were you last tested for any sexually transmitted infections (STIs)? Was it*…”	Within the last year/More than 1 year ago/Never/Don’t know/Prefer not to say	Within the last year or more than 1 year ago
	Abortion rate	*“How many of these pregnancies resulted in an abortion, medical or surgical for any reason?”*	Numeric count/Prefer not to say	% reporting ≥1 abortion (among ever pregnant/partnered)
**4) Sexual Health Outcomes**	HIV status	*“What was the result of your last HIV test? Was it…”*	Positive (you have HIV)/Negative (you do not have HIV)/You are still waiting for test results/Don’t know/Prefer not to say	Negative (you do not have HIV)
	STI treatment	*“Aside from HIV, when, if ever, did you last receive treatment for any sexually transmitted infection (STI)? This can be self-treatment or treatment from a doctor. Was it…”*	Within the last year/More than 1 year ago/Never/Don’t know/Prefer not to say	Within the last year or more than 1 year ago
	Unintended pregnancy	*“When you became pregnant with your last or current pregnancy, how much did you personally want to get pregnant? Did you…”*	Not want at all/Somewhat not want/Unsure/Somewhat want/Want very much/Prefer not to say	Not want at all, Somewhat not want, or Unsure
	Adolescent pregnancy	*“How old were you when you first gave birth?”(women/birthing parent)/”How old were you when your first biological child was born?” (men/non-birthing parent)*	Numeric age/Don’t know/Prefer not to say	% reporting first birth <20 years
	Sexual assault	*“Has another person ever forced or frightened you into doing something sexually that you did not want to do?”*	Yes/No/Don’t know/Prefer not to say	Yes
	Perceptions of safety	*“Outside your home, for example, at work or on the street, how safe do you typically feel from sexual assault?”*	Not at all safe/Somewhat unsafe/Somewhat safe/Don’t know/Prefer not to say	Not at all safe or Somewhat unsafe
	Sexual wantedness	*“Which statement applies best to you the most recent time you had sex? That is, the most recent time you had any sexual contact involving the genitals with another person. That may include oral sex, vaginal sex, anal sex, petting/fondling, rubbing genitals, etc.”*	I wanted it/I was unsure if I wanted it/I was forced or frightened into doing it/Don’t know/Prefer not to say	I wanted it
	Stillbirth rate	*“How many of these pregnancies resulted in stillbirth or baby born without heartbeat/breathing?”*	Numeric count/Don’t know/Prefer not to say	% reporting ≥1 stillbirth
	Fertility difficulties	*“Have you ever had a time lasting 1 year or longer when you and your partner were trying to get pregnant and it did not happen?”*	Yes/No/Don’t know/Prefer not to say	Yes

**Table 2.** Participant demographics.

	Total sample *N* (%)
	2555 (100)
Age	
18–24	241 (9.6)
25–39	700 (27.8)
40–59	862 (34.2)
60–74	596 (23.6)
75+	123 (4.8)
Missing	33 (1.3)
Sex	
Male	1101 (43.1)
Female	1431 (56.0)
Other	4 (.2)
Prefer not to say	11 (.4)
Missing	8 (0.3)
Gender	
Man	1094 (42.8)
Woman	1370 (53.6)
Other identity	77 (3.0)
Prefer not to say	10 (0.4)
Missing	4 (0.2)
Sexual Orientation	
Gay or lesbian	236 (9.2)
Straight	1806 (70.7)
Bisexual	408 (16.0)
Other	66 (2.6)
Prefer not to say	18 (0.7)
Don’t know	19 (0.7)
Missing	2 (0.1)
Marital Status	
Never married	1086 (42.5)
Married	1158 (45.3)
Separated	46 (1.8)
Divorced	211 (8.3)
Widowed	30 (1.2)
Prefer not to say	24 (0.9)
Race/Ethnicity	
American Indian/Alaska Native	80 (3.1)
Asian	147 (5.8)
Black or African American	283 (11.1)
Native Hawaiian or Pacific Islander	12 (0.5)
White	1942 (76.0)
Other	29 (1.1)
Hispanic/Latinx	280 (11.0)
Education	
>high school	82 (3.2)
high school	568 (22.3)
some college	567 (22.3)
college degree or higher	1331 (53.3)

**Table 3.** Estimates of sexual health in the U.S.

Objective 1. Increase knowledge, communication, and respectful attitudes								
	N	%	Men	%	Women	%	Other gender identities*	%
**Knowledge**								
**SHAPE:** (Yes) “Teaching sex education in school does not lead people to have sex earlier”	1998	**78.3**	777	**73**	1101	**81.9**	105	**87.5**
**Communication**								
**SHAPE PLUS:** (Completely) “Comfortable talking to your sexual partners about your sexual health?”	1235	**49.1**	526	**50.0**	636	**48.2**	64	**54.7**
**SHAPE PLUS: (**Completely) “Comfortable talking to any healthcare providers about your sexual health, relationships, and behaviors?”	785	**31.1**	359	**34.1**	395	**29.7**	27	**23.1**
**Attitudes**								
**SHAPE:** (Yes) “Sex between two consenting adult men is okay”	1781	**70.4**	706	**66.9**	949	**71.2**	113	**95.8**
**SHAPE:** (Yes) “Men naturally have more sexual needs than women”	625	**24.5**	264	**24.8**	346	**25.7**	10	**8.3**
**SHAPE:** (Yes) “It is okay for someone to have an abortion/terminate a pregnancy for ANY reason”	1222	**47.9**	467	**43.9**	653	**48.6**	92	**76.7**
**SHAPE:** (Strongly agree) “It is okay for someone to use a contraceptive method/family planning to avoid or delay pregnancy”	1984	**78.1**	756	**71.5**	1105	**82.3**	110	**91.7**
Objective 2. Increase healthy, responsible, and respectful sexual behaviors and relationships								
**SHAPE:** Sexual debut before age 18	984	**47.2**	405	**45.1**	537	**49.3**	37	**43.5**
**SHAPE:** No precautions/ protection at last sex (condoms, prep, pill etc.)	918	**59.8**	373	**56.6**	499	**62.4**	41	**59.4**
**SHAPE: (**Very/somewhat) pleasurable at last sex	1331	**87.1**	610	**93.1**	656	**82.3**	59	**85.5**
**SHAPE** (Very/somewhat) “satisfied have you been with your sex life in the last year”	1419	**55.6**	605	**56.8**	750	**55.8**	55	**45.8**
Objective 3. Increase use of high-quality, coordinated educational, clinical, and preventive services								
**SHAPE** Never been tested for HIV	1207	**49.6**	543	**52.8**	622	**48.4**	40	**34.8**
**SHAPE:** Never been tested for STI	1179	**46.6**	528	**49.7**	606	**45.0**	43	**36.8**
**SHAPE:** Abortion rate (of those who’ve ever been pregnant/gotten pregnant)	379	**29.2**	105	**23.4**	261	**31.9**	12	**38.7**
Objective 4. Decrease adverse health outcomes								
**SHAPE:** HIV Positive	44	**3.4**	40	**7.8**	2	**0.3**	1	**1.3**
**SHAPE:** Ever treated for an STI (combined ever and last year)	425	**16.8**	182	**17.1**	214	**15.9**	27	**23.1**
**SHAPE:** Unintended pregnancy (Not/somewhat wanted/unsure)	532	**40.1**	189	**40.0**	321	**38.9**	13	**81.3**
**SHAPE:** Adolescent pregnancy (age ≤19); asked to women/birthing people only	229	**18.9**	43	**10.5**	182	**23.3**	3	**13.6**
**SHAPE: Rape:** Ever forced or frightened you into doing something sexually	653	**27.3**	118	**11.7**	472	**37.0**	60	**56.1**
**SHAPE: Safety:** Feel Not at all/somewhat unsafe outside your home	392	**16.1**	60	**5.9**	285	**22.1**	45	**38.8**
**SHAPE:** Wantedness at last sex: “I wanted it”	1473	**89.2**	645	**91.1**	757	**87.4**	63	**90.0**
**SHAPE:** Stillbirth rate	44	**3.4**	18	**3.9**	23	**2.8**	2	**6.9**
**SHAPE**: Fertility difficulties	379	**15.2**	127	**12.1**	237	**17.8**	14	**12.1**

**Other gender identities include – transmen, transwomen and people who identified as “another term” at birth or “in another way” in terms of their current adult gender identity.

## Footnotes

1. Of note, this statement has not been approved by all UN member states.

2. Of note, the other gender identities category includes transmen, transwomen and people who identified as “another term” at birth or “in another way” in terms of their current adult gender identity.

3. Sensitivity analyses examining relationship context showed that most participants (~80%) reported their most recent sexual encounter occurred within an ongoing relationship (cohabiting/married or in a relationship but not cohabiting). These analyses revealed, for example that among women, non-use of protection at last sex was slightly higher within current relationships (65%) compared to the overall proportion (63%), whereas it was lower among women reporting casual or non-relationship encounters (51%), consistent with expectations that unprotected sex is more common in ongoing relationships than in casual encounters.
